# Combining Hyaluronic Acid and Amino Acids for Improved Healing of Post-Extraction Tooth Socket in Type 2 Diabetes Mellitus Subjects: A Randomized Clinical Trial

**DOI:** 10.3390/dj14020103

**Published:** 2026-02-11

**Authors:** Tiziana Ruggiero, Davide Camisassa, Marta Bezzi, Ettore Cogno, Benedetta Brugiafreddo, Vincenzo Nobile, Renato Pol, Ilaria Roato, Federico Mussano, Paolo Giacomo Arduino

**Affiliations:** 1 Department of Surgical Sciences, C.I.R. Dental School, University of Turin, Via Nizza 230, 10126 Turin, TO, Italy; 2 Department of Mechanical and Aerospace Engineering, Politecnico di Torino, Corso Duca degli Abruzzi 24, 10129 Turin, TO, Italy; 3 R&D Department, Complife Italia S.r.l., Via Mons. Angelini 21, 27028 San Martino Siccomario, PV, Italy

**Keywords:** type-2 diabetes mellitus, tooth socket, healing, tooth extraction, hyaluronic acid, amino acid

## Abstract

**Background/Objectives**: Conventional wound care often fails to address the complex pathology of diabetic wounds adequately. Research shows that hyaluronic acid and its derivatives promote tissue regeneration in the later stages of wound healing. We evaluated the efficacy of a novel topical formulation in promoting socket healing following post-extraction in patients with type-2 diabetes mellitus, by combining sodium hyaluronate and six amino acids involved in collagen synthesis. **Methods**: A single-center, two-arm randomized controlled trial was conducted in adults aged 18 and over with type 2 diabetes requiring extraction of at least one non-impacted tooth. Forty-four participants were randomized to receive either the intervention or no treatment. Primary outcomes included a modified Landry’s healing index and rate of socket closure. **Results**: Comparative analysis showed significantly improved healing index scores in the intervention group by day 7 and day 14 compared to control, with no improvements in the rate of socket closure. **Conclusions**: This research provides evidence on the therapeutic efficacy of the gel formulation under study in promoting wound healing of post-extraction sites in diabetic patients undergoing tooth extraction. Further research is needed to compare its efficacy with standard treatments and adjunct therapies.

## 1. Introduction

Diabetes mellitus is a progressive metabolic disorder that is associated with significant morbidity and mortality rates worldwide. The main types of diabetes include type 1 diabetes mellitus (T1DM), type 2 diabetes mellitus (T2DM), gestational diabetes mellitus (GDM), and other types that develop secondary to certain therapeutic interventions or are associated with specific genetic factors. In particular, T2DM accounts for most cases, approximately 96% globally [1,2,3].

Early projections anticipated a rise in global diabetes prevalence from 9.3% (463 million people) in 2019 to 10.2% (578 million) by 2030 and 10.9% (700 million) by 2045 [1]. However, by 2021, global prevalence had already surpassed the 2030 forecast, reaching 10.5% (537 million), and was forecast to reach 12.2% (783 million) by 2045 [2]. A more recent analysis by the Global Burden of Disease research group projects that diabetes cases will exceed 1.3 billion by 2050 [3].

This escalating prevalence in diabetes results from population aging and, more critically, from the global rise in obesity, which consistently exceeds projected figures annually [1,2,3]. Obesity is one of the strongest leading factors of type 2 diabetes, driving further progression of insulin resistance and chronic systemic inflammation, while impairing the function of pancreatic β-cells [1,2,3,4]. These pathophysiological processes interrelate, establishing a vicious cycle of metabolic dysregulation, eventually exacerbating the risk of vascular, immunologic, and endocrine complications [4].

The body’s ability to repair and regenerate tissues declines upon dysregulation of its systemic metabolism, contributing in this manner to macrovascular complications, including cerebrovascular, cardiovascular, and peripheral artery disease, but also in driving microscale accidents in tiny blood vessels, which can further lead to diabetic retinopathy, nephropathy, and neuropathy [3,4,5]. These secondary manifestations carry clinical significance for individuals with T2DM who undergo surgical procedures.

Patients who have diabetes often display delayed wound healing, either by primary or secondary intent, because of impaired tissue regeneration [6,7,8,9,10,11,12,13]. They are also at heightened risk of developing surgical site infections (SSIs) during surgical procedures, through mechanisms that extend beyond perioperative hyperglycemia [14]. The increase in perioperative risk supports the need for targeted management strategies in patients with diabetes [14,15]. Prognostic models consider the presence and severity of diabetes-related vascular complications to assess perioperative risk and predict adverse surgical outcomes [15,16,17].

Particularly, in oral care, several studies indicate that T2DM patients undergoing oral care procedures are also at risk of developing diabetes-derived complications with some added nuances [12,13,18,19]. The oral microenvironment in diabetes promotes chronic inflammation, which dysregulates growth factor signaling, and impairs regeneration of soft tissue within the context of the oral cavity; healing protracts while the risk of infection and need for antibiotic treatment of oral wound sites increases [8,9,10,11,12,13]. In the later stages of the healing process, dysregulated angiogenesis and altered macrophage polarization eventually contribute to compromised socket healing in post-extraction surgery [9,12,13,18,19,20,21,22].

Current therapies do not fully address the many complications encountered while managing diabetic wound sites [6,23,24,25]. Consequently, there is a need for alternative adjunctive therapies that target persistent inflammation, impaired angiogenesis, and disruption of immune cell responses during wound healing of diabetic patients. Researchers are exploring the use of adjunctive hyaluronic acid (HA)-based formulations designed to be delivered locally at wound sites. Some of these formulations show promise for patients living with diabetes, promoting tissue regeneration, and improving healing outcomes [26,27].

In oral surgery, hyaluronic acid has been reported to improve post-extraction wound healing [28,29,30]. In parallel, amino-acid-based approaches aim to support protein synthesis, particularly collagen, and immune balance in chronic wounds [23,24,28,29,30]. Consistent with this rationale, supplementing hyaluronic acid formulations with amino acids has been clinically investigated as a topical adjunct for oral wounds, with studies reporting improved healing-related outcomes and faster oral tissue regeneration [26,27,28,29]. Mechanistically, these formulations are thought to act through the role of hyaluronic acid in extracellular-matrix and tissue repair and its water-retention-barrier properties, alongside amino acids contributing to wound-healing processes such as collagen synthesis [23,26,27,29].

In patients with diabetes mellitus, delayed post-extraction healing remains a relevant clinical concern. Although several adjunctive approaches have been proposed, research in this area is ongoing and there is still no clear consensus on a standard adjunctive strategy of care [8,11,31]. Furthermore, hyaluronic acid has been investigated as a topical approach to support oral wound healing [27,28,29,32]; however, research into the possible benefits of applying such adjunctive treatments in post-extraction sockets in the context of diabetes is scarce, with only limited clinical evidence available to date [17,26].

In this clinical trial, we aimed to further investigate the efficacy of a topical gel formulation containing sodium hyaluronate (HA), supplemented with six synthetic amino acids (glycine, L-proline, L-leucine, L-lysine HCl, L-valine, L-alanine), in enhancing post tooth extraction healing in patients with T2DM compared with no treatment. This study seeks to provide further evidence regarding the clinical efficacy of topical HA-based adjunctive therapies in the diabetic post-extraction environment, where available data remain limited.

## 2. Materials and Methods

A total of 44 patients enrolled in this study. Participants were randomized into two groups; one received the designated topical intervention (n = 21), and the other group served as an untreated control (n = 23). This study assessed the healing index score and socket closure.

The study received ethical approval from the local ethics committee of the University of Turin (approval code 0100924 on 15 September 2022). This study was registered on ClinicalTrials.gov (Identifier: NCT05896319) with registration date 9 June 2023. This study was designed in accordance with the CONSORT 2010 guidelines for reporting of randomized controlled trials [33]. This manuscript addressed all the relevant items of the CONSORT 2025 checklist (Table S1). All participants provided the signed informed consent form. All procedures undertaken during the clinical phase adhered to the ethical standards outlined in the World Medical Association’s (WMA) Declaration of Helsinki and its subsequent amendments.

### 2.1. Study Design

This is a single-center, two-arm, randomized controlled trial conducted at the C.I.R. (Interdepartmental Research Center), Dental School, Section of Oral Surgery, Department of Surgical Sciences, University of Turin, between September 2023 and February 2024. Two-arm indicates that participants were randomized into two parallel groups: the topical gel intervention group and the control group receiving no adjunct treatment. The study population comprised patients diagnosed with type 2 diabetes mellitus who required extraction of at least one non-impacted tooth. Prior to enrolment, all participants were fully informed about the study procedures and provided signed written informed consent.

Patients requiring extraction of at least one non-impacted tooth were recruited and treated between September 2023 and December 2024. Subjects who met the following inclusion criteria were eligible for recruitment, including individuals aged 18 years or older; diagnosed with type 2 diabetes mellitus and a documented history of diabetes-related complications (nephropathy, neuropathy, retinopathy, cardiomyopathy, or peripheral vascular disease); and with a requirement for extraction of non-impacted teeth; having provided signed informed consent to participate in this study and with confirmed availability to attend follow-up visits. The exclusion criteria included the presence of platelet dysfunction or thrombocytopenia; ongoing corticosteroid therapy; current smokers; subjects who declined to participate in this study; poorly controlled diabetes mellitus; use of medications known to interfere with wound healing (e.g., corticosteroids, nonsteroidal anti-inflammatory drugs, chemotherapy drugs, immunosuppressants, biologic agents, systemic retinoids, vasoconstrictors); extractions requiring flap elevation; teeth requiring sectioning with burs; ankylosed teeth requiring bur use for extraction; and the occurrence of apical root fractures during the extraction procedure.

**Figure 1:**
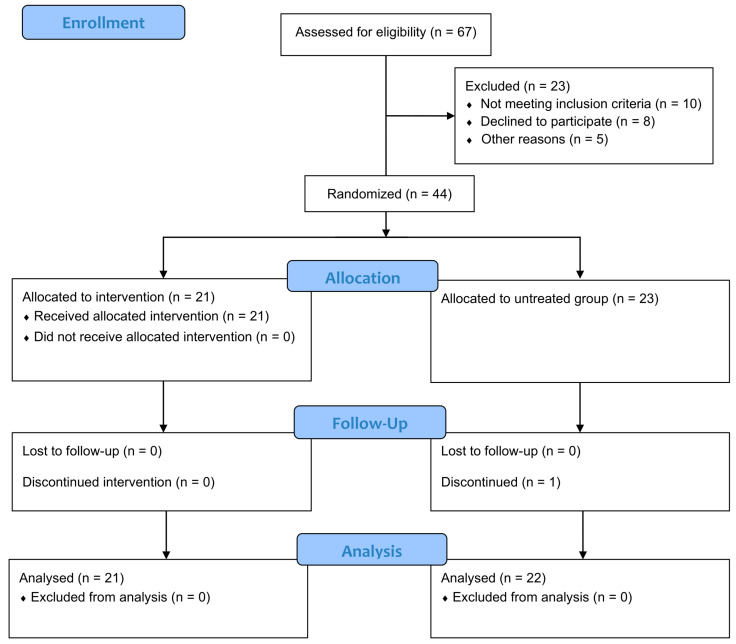
CONSORT 2025 Flow Diagram. Flow diagram of the progress through the phases of a randomized trial of two groups (that is, enrolment, intervention allocation, follow-up, and data analysis).

Tooth extractions were performed one at a time. Subsequently, extraction sites were randomly allocated to the test group (Aminogum/Aminogam 6^®^ gel, PROFESSIONAL DIETETICS S.p.A., Via Ciro Menotti, 1/A, 20129 Milan, Italy) and the control group (no treatment), using a computer-generated random sequence (SPSS version 24.0; SPSS Inc., Chicago, IL, USA). A total of 67 patients were screened, of whom 23 were excluded for the following reasons: 10 did not meet the inclusion criteria, 8 declined to participate, and 5 were excluded for other reasons (Figure 1).

### 2.2. Preoperative Data Collection

All participants received a professional oral hygiene session in advance of the tooth extraction. During the same visit, clinical and radiographic evaluations were performed to collect baseline data. Demographic characteristics recorded included gender, age, ethnic origin, body mass index (BMI), and smoking status. Periodontal health before surgery was performed using the periodontal screening record (PSR). Although plaque and bleeding indices were not documented before the professional cleaning, the PSR score is a validated measure of overall periodontal status. Diabetes-related variables were also recorded, comprising the duration of diabetes, glycemic status on the day of surgery (measured by blood glucose levels), glycated hemoglobin (HbA1c) levels, and end-organ disease score.

In addition, the preoperative status of the teeth scheduled for extraction was evaluated, including the number of roots (single- or multi-rooted), the presence of cavities, pulp vitality, any history of endodontic treatment, and the presence of any periapical lesions. The degree of extraction difficulty [17,31] was also recorded and classified into three categories based on space relative to mesiodistal distance, crown integrity, and root anatomy as reported by Ruggiero et al. [17]. Low indicated all parameters were of low difficulty with no more than one parameter of medium difficulty; medium indicated more than one parameter of medium difficulty but none of high difficulty; and high indicated one or more parameters of high difficulty [17,24,34]. While differences between upper and lower jaw or between single- and multi-rooted extractions were noted, the analysis focused on the effect of HA + AA treatment, which was applied uniformly across all extraction types. Therefore, separate subgroup analyses by jaw or number of roots were not performed, as the primary endpoint was the evaluation of the treatment effect rather than the influence of anatomical variation.

Systemic risk was evaluated as reported by Ruggiero et al. [17], based on the diagnostic and management criteria for diabetic patients provided by Mozzati et al. [31]. The classification system grouped patients into low/absent, moderate, and high systemic risk. Specifically, the systemic risk classification was based on a composite of parameters, including the end-organ disease score, time in years since diabetes diagnosis, typical blood glucose levels, and the presence of key clinical and treatment indicators [17,35]. These parameters were systematically collected to allow for inter- and intra-patient comparisons, while reducing the risk of bias related to baseline variability across patients at baseline.

### 2.3. Surgical Procedures and Postoperative Care

All the surgical procedures were carried out by the same experienced clinician, a specialist in oral surgery, who was blinded to the allocation of treatment and control groups. Preoperative and postoperative clinical assessments were conducted by trained examiners, who were also blinded to group allocation. Inter-examiner reliability was assessed using Cohen’s kappa statistic.

Tooth extractions were performed during the same surgical appointment. Local anesthesia was administered using either plexus or alveolar nerve block techniques, with 1.8 mL vials of 3% mepivacaine without vasoconstrictor (Opticain, Molteni Dental Srl, Firenze, Italy). Tooth extractions were conducted in a nontraumatic manner, avoiding elevation of a full-thickness mucoperiosteal flap to preserve both the alveolar bone crest and soft tissue integrity. Following tooth removal, the sockets were carefully debrided to eliminate any infected or granulation tissue, promoting wound healing. In cases where suturing was required due to underlying blood dyscrasias, nonabsorbable silk suture (Permahand 3/0, Ethicon, Somerville, NJ, USA) was applied using a crossed mattress (interrupted horizontal mattress) technique, chosen to ensure optimal wound edge approximation and hemostasis while minimizing tension on the tissue. All sockets were compressed using sterile gauze immediately post-extraction.

Participants were provided with standard postoperative instructions, including oral hygiene recommendations. In addition, patients in the treatment group received a 15 mL tube of the test product for topical application (see postoperative care below). No anti-inflammatory medications or antibiotics were routinely prescribed to avoid potential interference with the mechanism of action of the test product, due to the cytokine-inhibiting effect of nonsteroidal anti-inflammatory drugs (NSAIDs). Prescription of antibiotics postoperatively was recorded as a negative clinical outcome, as it indicated the presence of infectious complications.

The treatment group carried out postoperative care by topically applying the intervention, three times daily, at eight-hour intervals, for seven days. Postoperative care was self-administered following oral hygiene procedures, with patients instructed to refrain from swallowing, eating, or drinking for at least one-hour post-topical administration. Before use, patients were advised to wash their hands thoroughly. They were instructed to apply a layer of the gel directly onto the extraction site to fully cover the socket while gently massaging the area with a finger to ensure even distribution of the product and then compress the treated area with gauze.

### 2.4. Intervention Product

The product used comprises sodium hyaluronate and a combination of synthetic amino acids, which act as precursors in collagen synthesis (Aminogum/Aminogam 6^®^ gel, PROFESSIONAL DIETETICS S.p.A., Via Ciro Menotti, 1/A, 20129 Milan, Italy). Specifically, the formulation includes purified water, sodium hyaluronate, glycine, L-proline, L-leucine, L-lysine HCl, L-valine, L-alanine, methyl parahydroxybenzoate, propyl paraben, sorbitol, polyvinyl pyrrolidone, and sodium hydroxide.

### 2.5. Clinical Outcome Measures

Wound healing of post-extraction sockets was assessed using a modified version of Landry’s healing index as the primary outcome [17]. The Landry index was initially designed for wound healing by primary intention [36]. By contrast, post tooth extraction sockets heal, later progressing through granulation tissue development and subsequent epithelial coverage. To account for this difference, modifications were introduced to adapt the index for socket repair evaluation. In the modified Landry’s healing index, granulation tissue was interpreted as a favorable indicator of early connective tissue formation, rather than a negative feature as in the original scoring system [17,36]. This modified healing index incorporated four clinical parameters, tissue color, bleeding, granulation tissue, and suppuration. Each parameter was scored on a scale from 1 to 3, where higher values indicated poorer clinical presentation. The composite score or modified healing index was calculated as the sum of the individual scores for each parameter, ranging from 4 to 12, with higher scores indicating worse clinical outcomes. Specifically, tissue color scored 1 for fully pink gum (100%), 2 when ≤50% red, hyperemic, moving gum, 3 when ≥50% red, hyperemic, moving gum. Bleeding was registered as 1 if absent, 2 if induced by palpation, and 3 if spontaneous. Granulation tissue was scored as 1 if it was pink and firm (fine-grained in appearance), 2 if it appeared red and soft, and 3 if it was brittle, greenish or grayish. Suppuration was assessed by observing the presence of plaque and signs of alveolitis at the socket margins, where 1 indicated no accumulation of plaque on the margins, 2 visible plaque accumulation along the walls of the alveolus, and 3 suppuration or clinical signs of alveolitis (Table 1).

**Figure 2:**
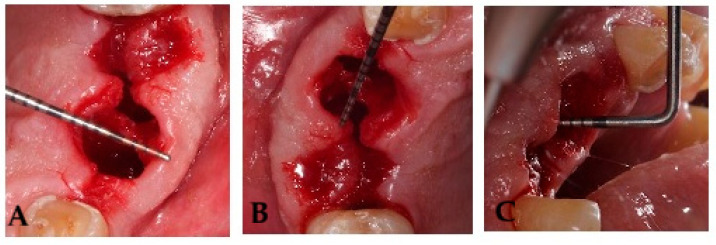
Images depicting linear measurements, expressed in millimeters, the maximum oral-vestibule OV diameter (**A**), the maximum mesiodistal MD diameter (**B**), and the maximum socket depth P (**C**).

Socket volume was calculated using three linear measurements, expressed in millimeters, the maximum oral-vestibular (OV) diameter, the maximum mesiodistal (MD) diameter, and the socket depth (SD) [17]. The mesiodistal diameter, oral-vestibular diameter, and socket depth were measured as previously described [17]. Measurements were taken at baseline (day 0) and on postoperative days 3, 7, and 14 with a Hu-Friedy PCPUNC 15 periodontal probe (Hu-Friedy, Chicago, IL, USA). Anatomical references were recorded for each tooth extraction to minimize measurement variability across time points (Figure 2); if complete socket healing had not been achieved by day 21, an additional assessment was scheduled.

### 2.6. Sample Size

The sample size for this study was calculated based on estimates derived from data presented in a previously published research article [17,32]. In brief, the sample size was calculated based on the primary outcome (wound healing at 14 days) assuming a mean difference of 1.0 mm and a standard deviation of 1.2 mm, with a significance level of 0.05 and 80% power. Accounting for a 10% dropout rate, 44 patients were enrolled.

### 2.7. Statistical Analysis

Continuous data were reported as mean ± standard deviation (SD) with 95% confidence intervals. Data normality was assessed using the Shapiro–Wilk test. Normally distributed variables were compared between groups using Student’s *t*-test, while non-normally distributed variables were analyzed using the Mann–Whitney U test. Categorical data were summarized as frequencies or percentages and analyzed using chi-square or Fisher’s exact tests as previously described by Ruggiero et al. [17]. All analyses were two-sided, and a *p* value < 0.05 was considered statistically significant.

## 3. Results

### 3.1. Baseline Characteristics

A total of 44 patients, were recruited and randomly allocated to either the treatment group (T6) or the control group (no treatment, C6). This study was conducted between September 2022 and February 2024. Participants were evenly distributed between the two study arms, with 21 individuals assigned to the designated topical intervention (T6), and 23 to the control group (C6). Baseline demographic and clinical characteristics are summarized in Table 2.

During the preoperative assessment, a statistically significant difference in age was observed between the two groups (*p* < 0.001), with the treatment group presenting a mean age of 78 ± 3.1 years, compared with 70 ± 6 years in the control group. Analysis of the body mass index (BMI) also revealed a significant difference (*p* < 0.05) with the treatment group displaying a mean BMI of 28.3 ± 5.8 kg/m^2^, versus 33.7 ± 9.4 kg/m^2^ in the control group. With respect to tobacco use, 13 participants in the treatment group and 8 participants in the control group were recorded as active smokers.

Regarding diabetes-related chronic complications, a total of 11 patients were diagnosed with retinopathy (5 in the treatment group, and 6 in the control group), 13 with nephropathy (10 in the treatment group, and 3 in the control group), and 22 with diabetic neuropathy (13 in the treatment group, and 9 in the control group). Diabetic cardiomyopathy was present in 43 patients (21 in the treatment group, and 22 in the control group), while peripheral vasculopathy was noted in 32 individuals (19 in the treatment group, and 13 in the control group). These clinical findings contributed to the overall systemic risk classification. Notably, all patients in the treatment group (n = 21) were classified as having high systemic risk, whereas in the control group, 7 were categorized as having high systemic risk, and 15 were categorized as having moderate systemic risk.

Assessment of periodontal health before surgery, using the periodontal screening record (PSR), revealed a statistically significant difference between the two arms (*p* < 0.01), with a mean PSR index of 3.5 ± 0.5 in the treatment group and 4.0 ± 0.5 in the control group. Preoperative surgery difficulty was classified as low, medium, or high, based on predefined criteria [17]. No cases were identified as having high surgical difficulty. A total of 25 participants were classified as medium-difficulty (16 in group T6, 9 in group C6), while 18 participants were classified as low-difficulty (5 in group T6, 13 in group C6) (Table 2). All enrolled participants completed this study, with no dropouts or losses to follow up. Consequently, all enrolled participants were included in the efficacy and safety analyses. The topical treatment was well tolerated across all subjects, with no adverse reactions or need for antibiotic therapy reported during the study period.

### 3.2. Modified Healing Index (mHI)

A comparative analysis of the modified healing index, conducted at multiple postoperative time points between day 0 (day of the extraction) and day 14 (at days 0 or D0, day 3 or D3, day 7 or D7, and day 14 or D14), revealed statistically significant differences in early tissue regeneration dynamics between the two study groups (Table 3). Healing progression within the post-extraction sockets was evaluated using a modified version of Landry’s healing index, incorporating four clinical parameters: tissue color, bleeding, granulation tissue, and suppuration. Each parameter was scored on a scale from 1 (optimal) to 3 (compromised) as described above.

At day 3 post-extraction, no statistically significant difference in healing index scores was observed between the two groups (*p* > 0.05), with the treatment and control groups presenting mean scores of 5.8 ± 2.1 and 6.0 ± 1.3, respectively. However, by day 7 (D7) and 14 (D14), statistically significant differences in healing outcomes emerged between the two study groups (*p* < 0.001). Specifically, at D7, the treatment group displayed a mean healing index of 4.0 ± 0.2 compared with 6.0 ± 1.4 in the control group. This trend persisted at day 14 (D14), at which point the group treated with the intervention continued to exhibit superior healing outcomes (4.0 ± 0.1) compared with the control group (5.0 ± 0.9) (Table 3).

### 3.3. Socket Measurements

To estimate the extent of socket closure, the subsequent analysis quantified the volumetric reduction in the post-extraction alveolus over time. To achieve this, the residual socket volume (RSV) was estimated using three principal linear measurements the maximum mesio-distal (MD) diameter, the maximum vestibulo-oral (VO) diameter, and the maximum probing depth (P). These parameters were recorded on day 0, 3, 7, and 14 to evaluate volumetric and morphological changes in the socket during the healing process. At T0 (day of tooth extraction), extraction socket measurements revealed statistically significant differences between the treatment and control groups in the mesio-distal (MD) and vestibulo-oral (VO) axes (*p* = 0.01). On average, patients in the treatment group presented with reduced MD and VO measurements, with mean values of 5.9 ± 2.7 mm and 6.0 ± 2.6 mm, respectively. By contrast, the control group displayed substantially larger dimensions, with the MD and VO parameters measuring 8.0 ± 2.7 mm and 8.0 ± 2.4 mm, respectively. No statistically significant variation in the socket probing depth (P) was observed between the groups at baseline (T6: 10.9 ± 3.3 mm; C6: 11.0 ± 3.5 mm), suggesting comparable vertical dimensions of the post-extraction alveolus at baseline (Table 4).

By day 3 post-extraction (D3), patients in the control group displayed mean values of 6.0 ± 2.4 mm for the MD axis, 5.0 ± 2.1 mm for the VO axis, and 9.0 ± 3.7 mm in socket depth, corresponding to an estimated residual socket volume (RSV) of 0.4 ± 0.2. Patients in the treatment arm consistently demonstrated a trend toward improved measurements along the MD and P axes, with mean values of 4.7 ± 2.0 mm and 7.6 ± 2.5 mm, respectively, and a statistically significantly lower vestibulo-oral (VO) distance of 3.8 ± 1.4 mm, resulting in a residual socket volume (RSV) of 0.5 ± 0.5, with no statistically significant difference in residual socket volume between the two groups (Table 4).

By day 7 post-extraction (D7), measurements across the three axes revealed that patients in the treatment arm had mean values of 3.5 ± 1.5 mm in the mesio-distal (MD), and 3.1 ± 1.4 mm in the vestibulo-oral (VO) dimension, and 5.8 ± 2.2 mm in probing depth (P), corresponding to a residual socket volume (RSV) of 0.3 ± 0.3. The control group exhibited mean measurements of 5.0 ± 2.1 mm (MD), 4.0 ± 2.1 mm (VO), and 6.0 ± 2.2 mm (P), with an associated RSV of 0.2 ± 0.1. At this stage of healing, the mesio-distal (MD) dimension was the only parameter to show a statistically significant difference between cohorts (*p* = 0.01) (Table 4).

Over time, both groups showed a progressive contraction in socket dimensions, consistent with ongoing post-extraction healing. By day 14 (D14), the treatment group demonstrated mean measurements of 2.8 ± 2.1 mm in the mesio-distal (MD), and 2.0 ± 1.5 mm in the vestibulo-oral (VO) axes, and 3.7 ± 2.5 mm in probing depth, corresponding to an estimated residual socket volume (RSV) of 0.1 ± 0.1. By comparison, the control group exhibited mean values of 3.0 ± 1.3 mm (MD), 3.0 ± 1.6 mm (VO), and 3.0 ± 2.5 mm (P), corresponding to an estimated RSV of 0.04 ± 0.03. At this time point, statistically significant differences were observed in both the VO dimension (*p* = 0.04) and RSV (*p* = 0.01), suggesting that while alveolar contraction over time was evident in both cohorts, the extent and morphological pattern of socket closure significantly diverged between groups (Table 4).

The topical gel was well tolerated, with no adverse reactions or need for antibiotic therapy.

## 4. Discussion

This clinical trial studied whether a gel containing sodium hyaluronate and six synthetic amino acids glycine, L-leucine, L-proline, L-lysine, L-valine, and L-alanine could improve healing of the socket post tooth extraction in patients with type 2 diabetes mellitus, a population frequently affected by protracted wound healing and compromised socket remodeling. A total of 44 patients with diabetes-related complications enrolled to assess the efficacy of the gel formulation in individuals at higher risk of impaired healing due to their systemic condition. A modified Landry index score was selected as the primary outcome to monitor soft tissue repair in post tooth extraction wounds [17].

Impaired healing of the tooth socket following extraction remains a significant burden to bear for patients with type 2 diabetes, who often exhibit reduced wound healing compared to non-diabetic or prediabetic individuals [19,37]. Interventions are frequently complicated by swelling and infection, making the search for alternative adjunctive strategies a priority in oral surgery [11,19]. Even though studies in humans point to some biomaterials, particularly hyaluronic acid and its derivatives, as critical components of formulations in enhancing soft tissue repair in diabetic patients, further research is needed to support these findings [17,26,31].

Patients treated with the intervention achieved statistically significant higher healing scores at day 7 and day 14 compared with controls (*p* < 0.001). Healing scores in the treatment group followed a course similar to what would normally be expected in non-diabetic individuals, whereas the control group required up to 21 days to achieve comparable scores [19,37]. Further, the intervention group achieved an optimal healing index score of 4 by day 7, whereas the control group had not reached this state by day 14 (remaining at 5), consistent with an earlier transition from the inflammatory/proliferative phases toward re-epithelialization and tissue maturation. These findings are consistent with meta-analysis and review articles that point to HA and its derivatives as promoters of soft tissue healing of diabetic ulcers in the absence of adverse events [38,39]. Randomized clinical trials also support the benefits of combining formulations with HA in achieving higher rates of healing in diabetic ulcers [40,41]. In oral surgery, a study using a split-mouth design in subjects with poorly controlled diabetes showed that locally applying HA after tooth extraction can lead to more rapid closure as well as earlier healing compared with untreated sites [17,30]. Beyond HA, formulations that combine HA with amino acids also show promising outcomes in wound healing. In oral soft tissue laser surgery, a gel containing sodium hyaluronate with glycine, L-leucine, L-proline, and L-lysine improved healing indices at day 7 compared with no treatment [28]. A similar formulation enhanced socket healing in patients with liver failure undergoing tooth extraction [42].

By contrast, residual socket volume (RSV), which reflects socket morphology dynamics, can diverge from soft tissue indices, especially in diabetic sockets where remodeling dynamics and baseline morphology may vary [8,24,34]. Even though both groups displayed progress in contraction profiles over time, the control group showed a greater reduction in RSV at day 14. The divergence in healing and socket contraction outcomes suggest that early soft tissue improvement does not translate into faster socket closure, consistent with complex asynchronous soft- and hard-tissue healing trajectories [8,18].

Thus, the results obtained in this study show that topically applying a combination of sodium hyaluronate plus pro-collagen synthesis amino acids facilitates soft tissue healing in patients with type 2 diabetes, even in the absence of marked volumetric advantages, while later driving tissue regeneration. From a mechanistic perspective, the formulation under study may support post-extraction healing by combining the role of hyaluronic acid in tissue repair with its water-retention/barrier properties, both of which can help maintain a moist and protected wound interface conducive to re-epithelialization [23,26,27,29], while the amino acid supplements may provide substrates supporting wound-healing processes such as protein and collagen synthesis, particularly relevant in the dysregulated healing environment observed in diabetes [23,24]. Because no further analyses of the biological processes underlying wound healing were performed, the mechanisms responsible for the observed divergence between improved clinical healing scores and unchanged socket-closure rates could not be determined.

## 5. Conclusions

Healing index results show that the intervention group exhibited significantly better healing outcomes by days 7 and 14 compared to the control. Socket dimension differences between the two groups over time were observed at individual time points across selected parameters, while socket volume contraction was significantly greater in the untreated group on day 14 only. Importantly, even though healing index results indicate a significantly faster progression of tissue repair in the treatment cohort compared to the control over time, socket morphology results suggest that far more complex dynamics are at work between socket morphology and tissue healing in post-extraction wounds.

## Figures and Tables

**Table 1:** Modified Healing Index (mHI) scoring system for post-extraction sockets. The mHI is based on four clinical parameters (tissue color, bleeding, granulation tissue, and suppuration), each scoring between 1 (best condition) and 3 (worst condition). Total score ranges from 4 (indicates excellent healing) to 12 (indicates poor healing).

Parameter	Score = 1	Score = 2	Score = 3
Tissue Color	Fully pink gum (100%)	≤50% red, hyperemic, mobile gum	≥50% red, hyperemic, mobile gum
Bleeding	Absent	Induced by palpation	Spontaneous
Granulation Tissue	Pink and firm (fine-grained appearance)	Red and soft	Brittle with greenish or graying discolouration
Suppuration	No plaque accumulation at socket margins	Visible plaque accumulation along the socket walls	Suppuration or clinical signs of alveolitis
Total Score	4 (excellent healing)	5–8 (moderate healing)	9–12 (poor healing)

**Table 2:** Demographic and clinical characteristics at baseline (BMI: body mass index; HbA1c: glycated hemoglobin; PSR: Periodontal Screening Record) (T6: Intervention Group; C6: Control Group). Data are presented as means ± standard deviation (SD) or numbers (percentages).

	Group T6 (n = 21)	Group C6 (n = 22)	*p*-Value
**Sex**			
Male	6 (28.6%)	20 (91%)	
Female	15 (71.5%)	2 (9.1%)	
**Ethnic origin**			
Caucasian	21 (100%)	22 (100%)	
**Age (years)**	78 ± 3.1	70 ± 6	*p* < 0.001
**Smokers**	13 (62%)	8 (36%)	
**BMI (kg/m^2^)**	28.3 ± 5.8	33.7 ± 9.4	*p* < 0.05
**HbA1c (%)**	6.8	7.5	
**End-organ disease score**			
Cardiomyopathy	21 (100%)	22 (100%)	
Nephropathy	10 (47.6%)	3 (13.6%)	
Neuropathy	13 (62%)	9 (40.9%)	
Peripheral vasculopathy	19 (90.5%)	13(59%)	
Retinopathy	5 (23.8%)	6 (27.3%)	
**Systemic risk**			
Low/Absent	0	0	
Moderate	0	15 (68%)	
High	21 (100%)	7 (32%)	
**Preoperative extraction difficulty levels**			
Low	5	13	
Medium	16	9	
High	0	0	
**PSR (index)**	3.5 ± 0.5	4.0 ± 0.5	*p* < 0.01

**Table 3:** Modified Healing Index (mHI) recorded at various postoperative time points (D3: day 3; D7: day 7; D14: day 14) (T6: Intervention Group; C6: Control Group). A score of 4 indicates optimal healing, while a score of 12 represents absence of or compromised healing.

Group	D3 (Mean ± SD)	D7 (Mean ± SD)	D14 (Mean ± SD)
T6 Group	6.0 ± 1.3	4.0 ± 0.2	4.0 ± 0.1
C6 Group	5.8 ± 2.1	6.0 ± 1.4	5.0 ± 0.9
*p*-value	>0.05	<0.001	<0.001

**Table 4:** Socket dimensions (MD: maximum mesio-distal distance; VO: maximum vestibulo-oral distance; P: maximum probing depth; RSV: residual socket volume) expressed in millimeters as mean ± SD (SD: standard deviation) at various postoperative time points (D0: Day 0; D3: Day 3; D7: Day 7; D14: Day 14) (T6: Intervention Group; C6: Control Group).

Group	D0	D3	D7	D14
**Maximum mesio-distal distance (MD) (mm, mean ± SD)**				
T6	5.9 ± 2.7	4.7 ± 2.0	3.5 ± 1.5	2.8 ± 2.1
C6	8.0 ± 2.7	6.0 ± 2.4	5.0 ± 2.1	3.0 ± 1.3
*p*-value	*p* = 0.01	----	*p* = 0.01	----
**Maximum vestibulo-oral distance (VO) (mm, mean ± SD)**				
T6	6.0 ± 2.6	3.8 ± 1.4	3.1 ± 1.4	2.0 ± 1.5
C6	8.0 ± 2.4	5.0 ± 2.1	4.0 ± 2.1	3.0 ± 1.6
*p*-value	*p* = 0.01	*p* = 0.03	----	*p* = 0.04
**Maximum probing depth (P) (mm, mean ± SD)**				
T6	10.9 ± 3.3	7.6 ± 2.5	5.8 ± 2.2	3.7 ± 2.5
C6	11.0 ± 3.5	9.0 ± 3.7	6.0 ± 2.2	3.0 ± 2.5
*p*-value	----	----	----	----
**Residual Socket Volume (RSV)**				
T6	----	0.5 ± 0.5	0.3 ± 0.3	0.1 ± 0.1
C6	----	0.4 ± 0.2	0.2 ± 0.1	0.04 ± 0.03
*p*-value	----	----	----	*p* = 0.01

## Data Availability

The data presented in this study are available on request from the corresponding author due to privacy restrictions.

## Supplementary Materials

The following supporting information can be downloaded at: https://www.mdpi.com/article/10.3390/dj14020103/s1, Table S1: CONSORT 2025 checklist [43].

## Abbreviations

The following abbreviations are used in this manuscript:

T2DM	Type 2 diabetes mellitus
HA	Hyaluronate
BMI	Body Mass Index
HbA1c	Glycated Hemoglobin
MD	Maximum mesio-distal distance
VO	Maximum vestibulo-oral distance
P	Maximum probing depth
RSV	Residual socket volume
